# Model Senescent Microglia Induce Disease Related Changes in α-Synuclein Expression and Activity

**DOI:** 10.3390/biom8030067

**Published:** 2018-08-01

**Authors:** Dafina M. Angelova, David R. Brown

**Affiliations:** Department of Biology and Biochemistry, University of Bath, Bath BA2 7AY, UK; D.M.Angelova@bath.ac.uk

**Keywords:** synuclein, tumor necrosis factor alpha, microglia, aging, iron, cytokines, tetramer

## Abstract

Aging is the most prominent risk factor for most neurodegenerative diseases. However, incorporating aging-related changes into models of neurodegeneration rarely occurs. One of the significant changes that occurs in the brain as we age is the shift in phenotype of the resident microglia population to one less able to respond to deleterious changes in the brain. These microglia are termed dystrophic microglia. In order to better model neurodegenerative diseases, we have developed a method to convert microglia into a senescent phenotype in vitro. Mouse microglia grown in high iron concentrations showed many characteristics of dystrophic microglia including, increased iron storage, increased expression of proteins, such as ferritin and the potassium channel, Kv1.3, increased reactive oxygen species production and cytokine release. We have applied this new model to the study of α-synuclein, a protein that is closely associated with a number of neurodegenerative diseases. We have shown that conditioned medium from our model dystrophic microglia increases α-synuclein transcription and expression via tumor necrosis factor alpha (TNFα) and mediated through nuclear factor kappa-light-chain-enhancer of activated B cells (NF-κB). The conditioned medium also decreases the formation of α-synuclein tetramers, associated ferrireductase activity, and increases aggregates of α-synuclein. The results suggest that we have developed an interesting new model of aged microglia and that factors, including TNFα released from dystrophic microglia could have a significant influence on the pathogenesis of α-synuclein related diseases.

## 1. Introduction

The study of neurodegenerative diseases has become a major incentive. This is due to their increased prevalence as the human population lives longer. This statement in itself immediately implies that a clearer understanding of these diseases will only come by greater insight into how the prevalence of these diseases is related to the aging process in the brain. Aging of the human brain is commonly associated with cognitive decline and it is the primary risk factor for the development of Alzheimer’s disease (AD) [1]. Despite these facts, the molecular biology of the aging brain has not been studied extensively. Some of the factors that change as we age include: the environment the neurons are exposed to and their transcriptome.

An example of the changing environment in the aging brain is the changes in the supporting cells in the brain, including microglia. Healthy microglia monitor their environment, phagocytosing debris, and releasing numerous molecules that can impact other cells [2]. They include hydrogen peroxide (H_2_O_2_), nitric oxide (NO), inflammatory cytokines, proteases, and neurotransmitters. Activated microglia can act as antigen presenting cells and activate T-cells. After an infection has been dealt with microglia can recruit cells that are involved in neuronal repair and secrete anti-inflammatory cytokines [3]. The idea of aging microglia stems from histological observations of healthy aged brains where the cells often develop dystrophic phenotypic characteristics [4]. Resting microglia have a ramified morphology with many fine processes extending from the cell body. Dystrophic microglia found in aging brains lose this fine process ramification. Dystrophic microglia often develop abnormally shaped processes with spheroidal swellings and cytoplasmic fragmentation (cytorrhexis) [5]. Dystrophic microglia have also been associated with the increased release of toxic ROS (reactive oxygen species) and inflammatory cytokines and impaired phagocytic ability [6,7,8]. The proinflammatory cytokines found to be released by dystrophic microglia include IL-6 (interleukin-6), TNF-α (tumor necrosis factor alpha), and IL-1β (interleukin 1-beta) [7]. However, one of the most unique changes observed in dystrophic microglia in the aging brain is the very high accumulation of iron, which is found to be stored in proteins, such as ferritin [9,10,11].

The presence of healthy glial cells is critically important to neuronal wellbeing. Microglia maintain homeostasis in the healthy brain and fight infection, when it is present, through a complicated system of signalling molecules [12]. The importance of microglia to neurons is supported by higher incidence of dystrophic microglia and microglial apoptosis in Alzheimer’s disease [13]. The inflammation of the nervous system in neurodegenerative disease was thought to be due to activated microglia. However, low, but sustained, release of inflammatory factors and impaired neuroprotective ability of microglia seen in neurodegeneration could be due to dystrophic changes instead [14]. 

The cytosolic protein alpha-synuclein (α-syn) is associated with a range of neurodegenerative diseases, including Parkinson’s disease (PD) Dementia with Lewy bodies (DLB), the Lewy body variant of Alzheimer’s disease, and multiple system atrophy. Aggregated α-syn is concentrated in structures termed Lewy bodies and Lewy neurites that are associated with the synucleinopathies [15,16]. Extracellular α-syn is present as aggregates in both the substantia nigra of PD patients [16] and senile plaques of AD brains in the form of the non-Aβ component (NAC) [17,18]. Clear links between α-syn and neurodegeneration have been found. Neuronal cell loss and Lewy body-like inclusions occur in animal models overexpressing α-syn [19] and the rescue of dopaminergic cells from death occurs following the down-regulation of α-syn expression in the substantia nigra of a Parkinson’s disease rat model [20]. 

In PD there has been discussion of the possible involvement of microglia [21] and experiments with rodent PD models have shown that microglial activation can cause PD-like symptoms [22]. Parkinsonian changes in primate brains induced by manganese have been shown to be accompanied by dystrophic changes in microglia [23]. Additionally, dystrophic microglia have been identified in DLB [24]. Age-related changes in microglia have also been suggested to play a role in Parkinson’s disease [25].

In the current work, we establish iron overload as a mechanism to switch microglial phenotype to one that has many of the characteristics of senescent microglia. Iron overload was achieved by growing microglia in high concentrations of iron. We also show that iron overloaded (iron-fed) microglia release factors, including increased levels of the cytokine TNFα that caused an increased expression of α-syn, altered its activity, and increased its aggregation. We argue that such a model of senescent microglia could be utilized to improve models for the study of neurodegenerative diseases by the incorporation of this age-related change. 

## 2. Materials and Methods

Reagents were purchased from Sigma-Aldrich (Poole, UK) unless otherwise stated.

### 2.1. Cell Culture

SH-SY5Y (human neuroblastoma) cells were cultured in 45% DMEM (Dulbecco’s modified Eagle’s medium)/45% Ham’s F12 (LONZA, Basel, Switzerland) supplemented with 10% FBS (foetal bovine serum) (Labtech, Heathfield, UK), and 1% penicillin/streptomycin. Cells were maintained at 5 × 10^6^/75 cm^2^ at 37 °C and 5% CO_2_ in a humidified incubator. The neuronal status of SH-SY5Y cells was monitored by reverse transcription polymerase chain reaction (RT-PCR) with primers for tyrosine hydroxylase (TH), dopamine transporter (DAT), and vesicle monoamine transporter 2 (VMAT2). 

Cell lines derived from SH-SY5Y cells and overexpressing α-syn were developed by stable transfection of plasmids (pCDNA3.1) containing the open reading frame (ORF) of the protein using Fugene (Promega, Southampton, UK). The cell line generated was as previously described [26].

The microglial cell line used in this study was C8B4 (CRL2540, American Type Culture Collections (ATCC), mouse). The cell line was grown in DMEM with 10% FBS, and 1% penicillin/streptomycin. Primary microglial cells were also used for some experiments and were prepared, as previously described [27,28]. Mouse primary microglia were generated from new born mice (balb/c). Mice were sacrificed according to a schedule 1 procedure. The procedure was endorsed by the Animal Welfare and Ethics Review Board of the University of Bath and was in accordance with the Guidance on the operation of the Animals (Scientific Procedures) Act 1986. Brains of the mice were dissected and dissociated by both trypsinization and mild mechanical disruption. Cultures were seeded with cells from 4–5 brains per 75 cm^2^ flask. Cells were grown at 37 °C and 5% CO_2_ for two weeks in the same medium as the microglial cell lines. The resultant cultures were mixed glial cultures composed of predominantly astrocytes and microglia. Microglia were separated from other glia by partial trypsinization, which causes the detachment of the overlying astrocytes [28]. The remaining cells were then collected by further trypsinization and panning. Microglia have higher adhesiveness and most remaining contaminating cells could be removed after 20 min of plating. The remaining adherent cells were microglia. Routine verification of purity was determined either by phenotype or by staining with ferritin.

Microglia were cultured in high iron to induce a dystrophic/senescent phenotype. Microglial cell lines were grown in medium containing in 500 μM ferric ammonium citrate for a minimum of two weeks. Ferric ammonium citrate was prepared in deionized water at 25 mM and filtered (0.22 μm filter) before addition to cultures. Cell lines were maintained under these conditions until being used for experiments. Primary microglia were treated with 500 μM ferric ammonium citrate while in mixed glial cultures before purification. Prior to experiments control and iron-fed primary microglia were isolated from the mixed cultures and plated at 30% density in six well trays. Conditioned medium was generated from microglial cell lines and primary microglia plated at 30% density. The medium used for the generation of conditioned medium was DMEM supplemented with B27 without antioxidants (Gibco, ThermoFisher, Waltham, MA, USA) and 1% pen/strep. Iron-fed microglia were extensively washed to remove excess iron before medium was applied. Conditioned medium was collected after 48 h, centrifuged at 170× *g* to remove debris, and filtered with a 0.45 μm filter before use in experiments. The cell density that was used to generate conditioned medium was confirmed by lysing the cells after collecting the conditioned medium and measuring the protein concentration using a Bradford assay (Bio-Rad, Watford, UK). The data was normalized to this value.

### 2.2. Western Blotting

Cells were lysed in PBS with 0.5% Igepal CA-630 and ‘complete’ protease inhibitor cocktail (Roche, Welwyn Garden City, UK), sonicated 20 s on ice, incubated on ice for 20 min, and centrifuged 10,000× *g* for 5 min to remove insoluble membranes. Protein concentration was determined with a Bradford protein assay (Bio-Rad), according to the manufacturer’s instructions. Protein concentrations were normalized and the samples were boiled for 5 min with 1× Laemmli sodium dodecyl sulfate-polyacrylamide gel electrophoresis (SDS-PAGE) buffer. Samples were loaded into either a 10% or a 14% (depending on molecular weight of the protein) acrylamide SDS-PAGE gel, with a buffer of Tris (250 mM) + glycine (1.92 M) + sodium dodecyl sulfate (SDS) (0.1% *w*/*v*), run at 250 V/35 mA for 45–60 min. Separated proteins were transferred to a polyvinylidene difluoride (PVDF) membrane by a semi-dry transfer apparatus, run at 25 V/100 mA for 1.5 h. Membranes were blocked in 5% *w*/*v* non-fat milk powder in TBS-T (Tris-buffered saline + 0.05% tween 20) for one hour, incubated with primary antibody for 1–2 h or overnight, and washed 3 × 15 min in TBS-T. Membranes were blocked again and incubated with horseradish peroxidase-conjugated secondary antibody for 1 h. A further 3 × 15 min washes were performed, and the membranes developed with Luminata Crescendo or Luminata Forte ECL substrate (Millipore, Watford, UK) and imaged with a Fusion SLCCD imaging system (Vilber Lourmat, Collégien, France).

Rabbit monoclonal anti-α-synuclein (MJFR1, Abcam, Cambridge, UK) was used for human α-synuclein detection at a dilution of 1:4000. Mouse monoclonal anti-α-tubulin (T5186, Sigma) was used at a dilution of 1:10,000. Anti-l-ferritin mouse monoclonal (SC-25616, Santa Cruz, Dallas, TX, USA) was used at 1:5000, anti-Kv1.3 rabbit polyclonal was used at 1:400 (APC101, Alomone, Jerusalem, Israel), and anti- Glyceraldehyde 3-phosphate dehydrogenase (GAPDH) mouse monoclonal was used at 1:2000 (6C5, Abcam). Densitometry was carried out using ImageJ. 

### 2.3. Detection of α-Synuclein Tetramers and Aggregates and Ferriductase Assay

Measurement of ferrireductase activity was performed on extracts of SH-SY5Y cells transfected to overexpress human α-syn. The stable cell line and the method to measure the activity were as previously reported [29]. Tetramers were detected by crosslinking the extracts with the crosslinker, disuccinimidyl suberate (DSS) (Sigma), according to the manufacturer’s instructions and as previously described [29]. Crosslinked species were detected with western blot, as described above, for non-crosslinked α-syn. High molecular weight aggregates of α-syn were detected, as previously described in cross linked samples of SH-SY5Y cells overexpressing α-syn [26].

### 2.4. Iron Assay

The levels of total iron in C8B4 cells and conditioned medium were determined using a commercial assay (Abcam) and following the manufacturer’s instructions. Cellular iron content was determined from four confluent T25 flasks and normalized to total protein content determined by the Bradford assay. Conditioned medium was concentrated 10-fold using a Speedvac Concentrator (Savant, ThermoFisher).

### 2.5. Reactive Oxygen Species Assay

The method to determine reactive oxygen species was based on Uy et al., 2011 [30]. Microglia (both control and iron-fed) were re-plated to equivalent density. The medium of the cells was replaced with serum free medium (DMEM) supplemented with B27 without antioxidants. After 24 h, the medium was collected from the microglia and then cleared by centrifugation at 10,000× *g* for one min. Control medium (50 µL) was used as a blank. The assay was executed in a white 96 well plate. 10, 20 30, 40, and 50 µL of the conditioned medium was plated into the plate and made up to 100 µL with distilled water. The assay was initiated by the addition of 100 µL of chemiluminescence reagent (Luminata Crescendo, Millipore). The plate was incubated in the dark for five min before reading. Measurements of luminescence were performed using the Omega FLUOstar plate reader (MBG, Labtech GmbH, Offenburg, Germany) at 482 nm. Background was subtracted for all values.

### 2.6. Cytokine Quantitation

Analysis of cytokines present in conditioned medium was determined while using the Meso Scale Discovery (MSD, Rockville, MD, USA) Sector S 600 multiplex imager system. The plate used for the analysis was the V-Plex Proinflammatory mouse kit 1 (MSD). This kit allowed for the assessment of the following cytokines: TNF-α, IFN-γ (interferon gamma), IL-1β, IL-2, IL-4, IL-5, IL6 (interleukin-6), IL-10 (interleukin-10), IL-12p70 (interleukin-12p70), and KC/GRO (keratinocyte chemoattractant/growth-regulated oncogene). Conditioned medium was prepared as described above and filtered through a 0.22 μm filter before applying to the plate as per the manufacturer’s instructions. Concentrations were determined by comparison to a standard curve for each individual cytokine. The values were adjusted for plating density by assessing the protein content of the cells used to generate the conditioned medium while using the Bradford assay and dividing the cytokine concentration by the protein concentration.

### 2.7. Promoter Assays

SNCA Promoter fragments and assay conditions were as previously described [31]. SH-SY5Y cells were performed in 24-well plates seeded at 5 × 10^4^ cells/well 24 h prior to transfection. Transfections of promoter constructs in pGL Basic (with firefly luciferase activity) were performed using FuGENE HD transfection reagent (Promega), as per manufacturer’s instructions. To control for variation in transfection efficiency among replicates, promoter constructs were co-transfected with the Renilla luciferase vector, pRL-TK (Promega). At 24 h post transfection, SH-SY5Y cells were harvested and firefly and Renilla luciferase chemiluminescence were measured while using the Dual-Luciferase Reporter Assay System (Promega) in a BMG FLUOstar Omega plate reader (BMG Labtech GmbH, Offenburg, Germany). Luciferase activity was calculated as the ratio of firefly to Renilla luciferase activity.

### 2.8. Proliferation Assay

Proliferation of C8B4 microglia was assessed using the Abcam BrdU proliferation ELISA kit (ab126556, Abcam) according the manufacturer’s instructions. Both untreated and iron-fed C8B4 microglia were plated at equal density ranging from 2000 to 20,000 cells per well in 96-well trays. The cells were exposed to BrdU (bromodeoxyuridine) for 24 h prior to starting the assay. Absorbance was measured at 450 nm following the colorimetric assay.

### 2.9. Toxicity Assay

The level of cell death during treatment of C8B4 cells with 500 μM ferric ammonium citrate was measured using the Roche Cell Death Detection ELISA^PLUS^ kit. The procedure was carried out following the manufacturer’s instructions. C8B4 cells were plated into 24 well trays at low density (10%). The treatment was carried out over ten days (medium changed every two days). Measurements of cell death were carried at 1, 2, 4, 6, 8, and 10 days of treatment. The level of cell death in iron-fed cells was compared to that of untreated cells as a percentage.

### 2.10. Statistics

All of the statistics were carried out in Microsoft Excel. Statistical analyses were conducted using a two-tailed Student’s *t* test, with statistical significance at *p*-value of <0.05. Data are expressed as the mean ± standard error (S.E.M.).

## 3. Results

### 3.1. Generation and Characterisation of a Senescent Phenotype in Microglia

We hypothesized that the senescent microglial phenotype might be related to changes in iron storage. Iron (particularly Fe(II)) causes damage to macromolecules [32] and simply increasing the levels of iron that is stored may be sufficient to induce changes seen in the aged phenotype. For this reason, we grew the murine microglial cell line C8B4 in 500 μm ferric ammonium citrate for at least two weeks. The C8B4 cell line was used because it allowed for the production of large numbers of microglia to facilitate the study. C8B4 cells grown under these conditions will be referred to as iron-fed microglia. 

**Figure 1:**
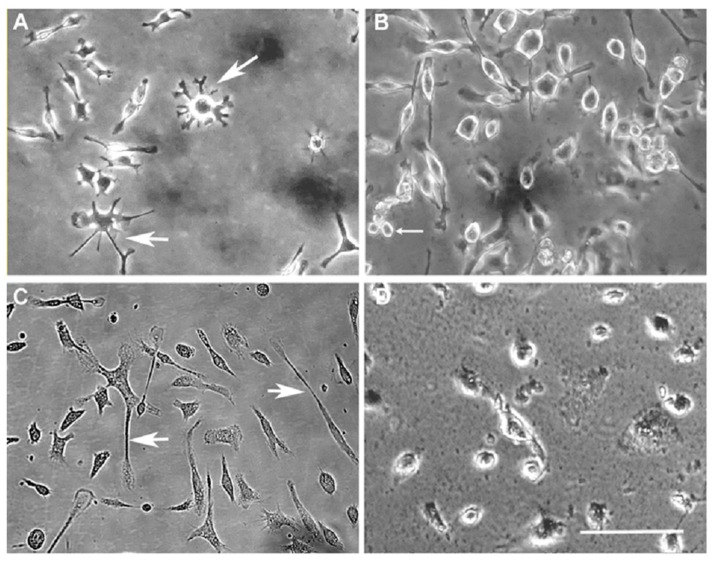
Phenotype of iron-fed microglia. Photomicrographs of microglia were prepared from both C8B4 (**A**,**B**) and primary mouse microglia (**C**,**D**) in culture. The microglia were either grown in control conditions (**A**,**C**) or grown in 500 μM ferric ammonium citrate (iron-fed, **B**,**D**). Both C8B4 and primary microglia showed clear phenotypic changes when iron-fed. These include loss of the projections seen in control cells (large arrow head) and the appearance of cytoplasmic fragmentation (small arrow). Scale bare = 50 μm.

Iron-fed C8B4 microglia showed morphological changes that are similar to dystrophic microglia. Under normal culture conditions (Figure 1A) C8B4 cells showed a small cell body with multiple projections and frequent ramification. In contrast, iron-fed microglia (Figure 1B) showed no ramification and little to no projections and frequent membrane fragmentation. We also examined primary microglia for evidence of similar phenotypic changes. While primary microglia under normal culture conditions showed projections (Figure 1C), iron-fed primary microglia were largely ameboid with no projections at all (Figure 1D).

**Figure 2:**
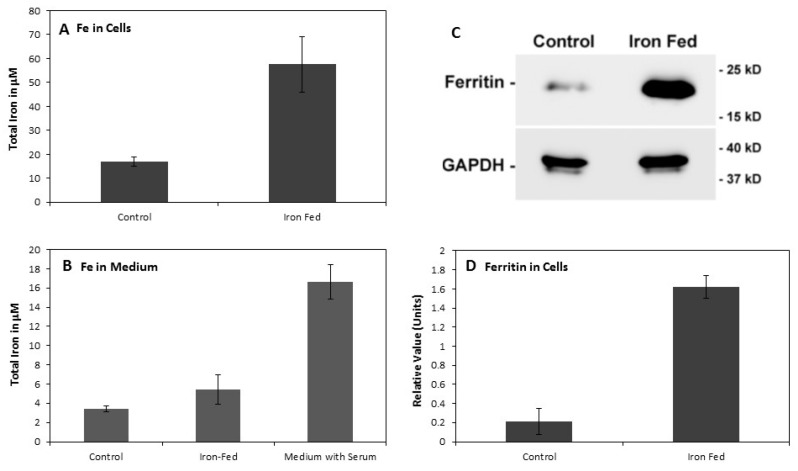
Changes in iron storage C8B4 microglia that had been grown in the presence of high ferric ammonium citrate were analysed for changes in iron content, iron release, and ferritin expression. (**A**) We used a commercial kit to analyse the iron content of microglia grown in 500 μM iron for at least two weeks. Microglia were washed three times before analysis to remove excess iron from the medium. Extracts were then made from the microglia and the iron content determined for both control and iron-fed microglia. The analysis showed a high and significant (*p* < 0.05) change in the iron within C8B4. (**B**) The level of iron released into the culture medium was assessed similarly. Conditioned medium (B27 supplemented DMEM was produced from control and iron-fed C8B4 microglia by exposing them for 24 h. The medium was concentrated 10-fold by lyophilisation and the concentration compared to serum containing medium used to maintain the C8B4 microglia. While iron-fed microglia showed no significant increase in iron released in the medium compared to controls (*p* > 0.05) this concentration was significantly lower than found in normal serum supplemented conditioned medium (*p* < 0.05). (**C**) The levels of L-ferritin were determined in iron-fed microglia by western blot. Protein extracts were prepared and equal amounts of protein from control and iron-fed microglia were run on a 10% PAGE gel, transferred to a membrane and L-ferritin was detected on the membrane with a specific antibody. Another antibody was used to detect GAPDH to confirm equivalent protein loading. (**D**) The densitometric quantitation following normalization to GAPDH levels showed there was a major and significant (*p* < 0.05) increase in L-ferritin expression levels in iron-fed microglia. Shown are the mean and S.E.M. of four experiments for each part.

After two weeks of treatment, iron-fed microglia were analysed for changes in both iron content and the iron storage protein ferritin. Cells were washed free of serum and iron and left for 24 h in serum free medium before harvesting in serum free medium. Analysis of the iron content of C8B4 microglia showed that there was an approximate five-fold increase in the amount of iron that is present in the cells when iron-fed (Figure 2A). Conditioned medium generated from iron-fed microglia showed no significant increase in the amount of iron released (Figure 2B). Also, the level of iron released/present in the medium conditioned by the microglia was lower than the amount if iron present in the medium that these cells were normally cultured in (with 10% serum). Iron is mostly stored within the protein ferritin in microglia and increased ferritin expression is associated with a change to a dystrophic phenotype [9]. We assessed the level of L-ferritin in iron-fed C8B4 by western blot (Figure 2C,D). Iron-fed microglia showed a very high (five-fold) increase in the level of ferritin expressed. These results show that iron-fed microglia have a similar pattern of increased iron storage as dystrophic microglia.

### 3.2. Proliferation and Cell Death

**Figure 3:**
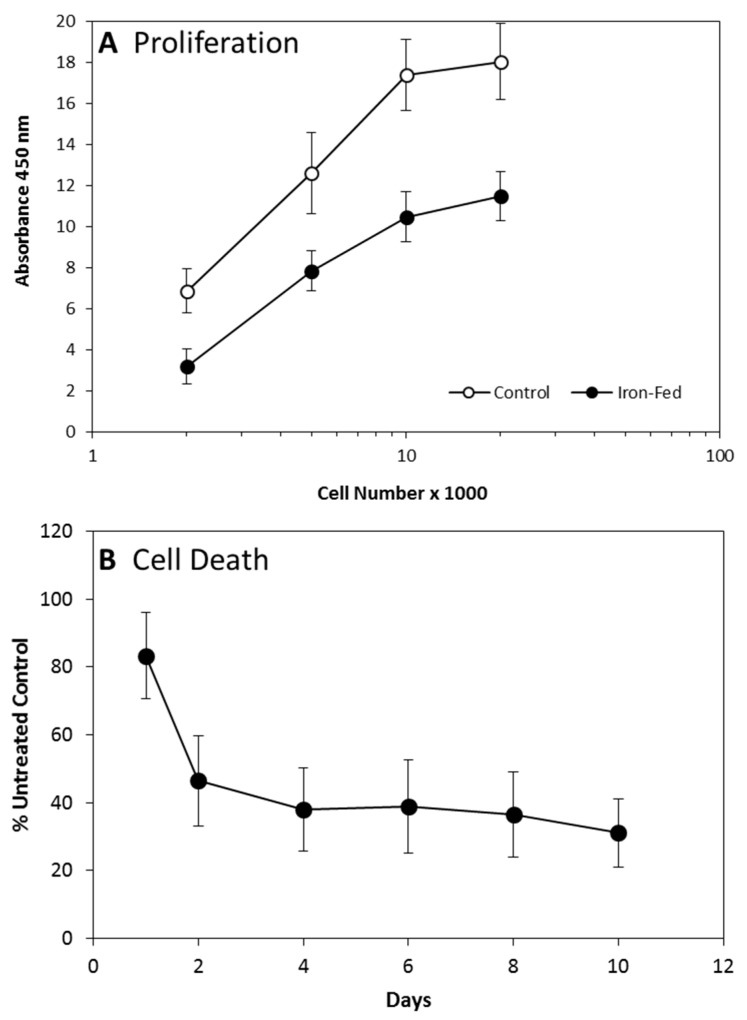
Proliferation and Cell Death (**A**) The rate of proliferation of C8B4 microglia was assessed using a bromodeoxyuridine (BrdU) based ELISA kit. Both control and iron-fed microglia were plated into a 96 well plate at a range of densities and grown overnight. BrdU was then added for a further 16 h before the ELISA assay was used to assess incorporation levels. The level of incorporation was assessed by a colorimetric assay with a read out at 450 nm. Iron-fed microglia showed significantly lower levels of proliferation at all plating densities other than the highest (*p* < 0.05). Shown are the mean and S.E.M. of four separate experiments. (**B**) The level of cell death in C8B4 cells was assessed during their initial treatment with 500 μM ferric ammonium citrate. C8B4 cells were plated in 24-well trays at low density (20% confluency). The cells were then treated with iron for up to 10 days. The level of cell death was assessed using a commercial ELISA kit that determines the levels of histone associated DNA fragments in the cytoplasmic fraction. The results showed a significantly lower level of cell death in cells treated with iron for 2–10 days (*p* < 0.05). Only cells treated for one day showed no significant difference to the untreated cells (*p* > 0.05). Shown are the mean and S.E.M. of four separate experiments.

We determined the impact of the iron-fed phenotype on the rate of proliferation of C8B4 microglia. We used an ELISA assay based on BrdU (bromodeoxyuridine) incorporation. Both control and iron-fed microglia were plated at a range of densities in serum free medium supplemented with B27 and exposed to BrdU overnight. The level of incorporation of the label was then assessed according to the manufacturer’s instructions. The level of BrdU incorporation was significantly different between iron-fed and control microglia at all plating densities except for the highest (Figure 3A). At the highest density, cellular crowding probably impacted the rate of proliferation. However, the results suggest that iron-fed microglia proliferate at a significantly lower rate than control microglia.

We also wished to determine if C8B4 microglia experience cell death during the treatment that results in the iron-fed phenotype. C8B4 microglia were exposed to 500 μM ferric ammonium citrate for 10 days. Cell death was measured using an ELISA kit that assesses cytoplasmic histone-associated DNA fragments. The assessment was carried out at 1, 2, 4, 6, 8, and 10 days of treatment and compared to the value that was obtained from the untreated control as a percentage. After one day of treatment there was no significant difference to the untreated control (Figure 3B). However, on all subsequent days the level of cell death detected fell significantly below that of the untreated control and the one-day treatment. After day 2, there was no significant change in the level of cell death. These results suggest that the treatment with iron had no significant toxic effect, and in fact there was a significant reduction in spontaneous cell death. However, it should be kept in mind that there was also a significant reduction in cell proliferation in iron-fed microglia, which would suggest that the difference observed is a result of reduced cell number rather than reduced spontaneous cell death.

### 3.3. Altered Protein Expression in Iron-Fed Microglia

**Figure 4:**
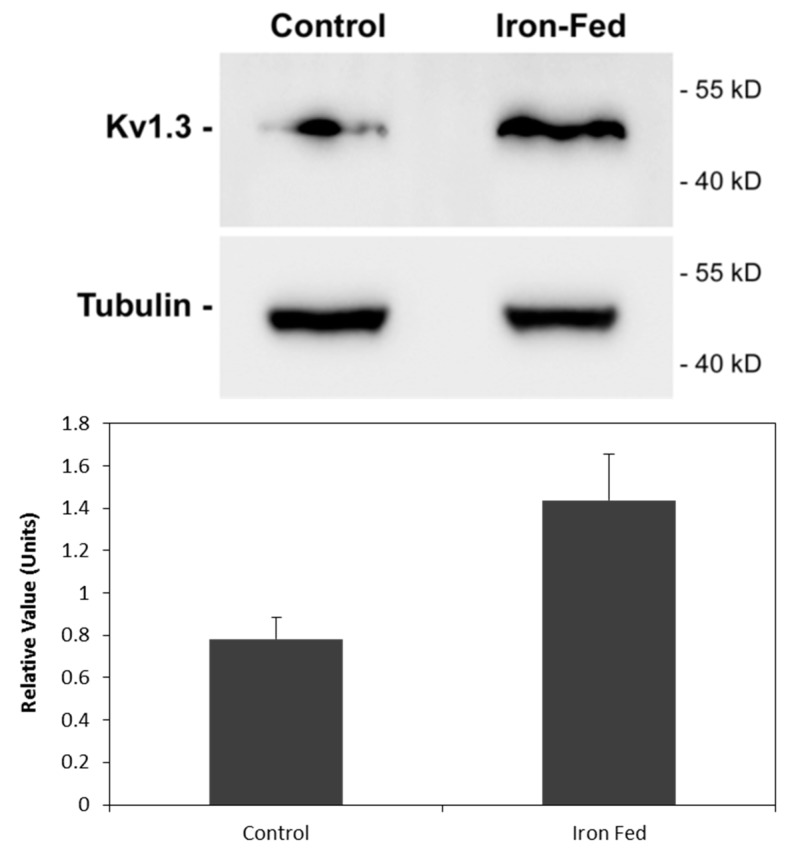
Expression of the potassium channel Kv1.3. Protein extracts were prepared from control and iron-fed C8B4 microglia. Western blot analysis was carried out to determine the level of Kv1.3 in the microglia. Bands for the protein were observed in both control and iron-fed microglia. Levels of tubulin were also determined to verify protein loading. The results showed a significant (*p* < 0.05) elevation of Kv1.3 in iron-fed microglia. Shown are the mean and S.E.M. of four separate experiments.

The characterization of the senescent microglia phenotype by altered protein expression remains elusive, as there are currently no agreed changes that conclusively define microglia as being dystrophic. However, a recent study suggested that aged microglia show an increase in the potassium channel Kv1.3 [33]. We analysed the expression of Kv1.3 by iron-fed C8B4 using western blotting and found a significant increase relative to control cells (Figure 4). This suggests that similar to aged microglia, iron-fed microglia expressed increased levels of this potassium channel.

### 3.4. Reactive Oxygen Species Production

**Figure 5:**
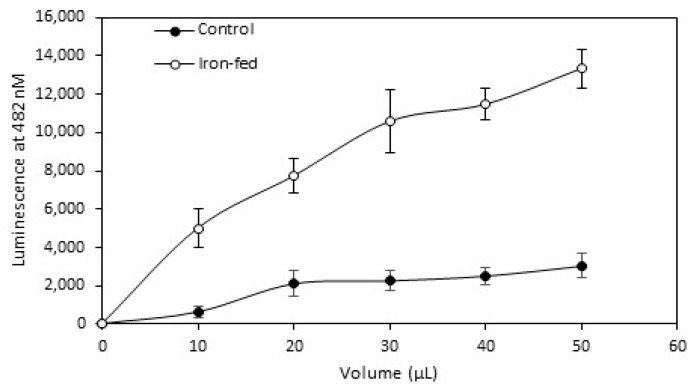
Reactive oxygen species (ROS). Control and iron-fed C8B4 microglia plated at equal density were exposed to serum free medium (without phenol red) for 24 h. Medium was collected and centrifuged and assessed for ROS using a luminescence assay with detection at 482 nm. Medium from iron-fed microglia showed significantly (*p* < 0.05) higher levels of ROS than control microglia. Shown are the mean and S.E.M. of five separate experiments.

One of the most common changes in microglia is an increased production of (ROS)upon activation. We wished to assess whether iron-fed microglia also show a change in ROS production. We used a simple spectrophotometric assay to assess the change in ROS generated by iron-fed C8B4 microglia compared to control microglia. We generated conditioned medium from both kinds of microglia plated at an equal density by exposure of the microglia to serum free medium with the B27 antioxidant free supplement overnight. We applied increasing volumes of the medium to the assay and measured the ROS generated. Iron-fed microglia produced significantly higher levels of ROS than control microglia (Figure 5). This suggests that iron-fed microglia show some aspects of an activated phenotype similarly to aged microglia.

### 3.5. Cytokine Expression

Cytokines produced by microglia constitute the single most significant way that microglia interact with other cells. Therefore, changes in cytokine expression would indicate a potential mechanism by which dystrophic microglia could influence neuronal activity. A sensitive assay system was used to measure the level of multiple proinflammatory cytokines produced by both C8B4 and primary mouse microglia in culture. We analysed 12 different cytokines secreted into conditioned medium. However, in both primary and C8B4 microglia a number of tested cytokines had expression levels below the detection limits of the assay (IFNγ, IL-2, IL-4, IL-5, IL-8, IL-12p70, and IL-13). KC/GRO was significantly elevated in iron fed primary microglia but could not be detected in C8B4 microglia (Table 1). IL-1β, IL-6, IL-10, and TNFα were detected in the conditioned medium of both cell types, but the changes that were observed in the two cell types were only the same for TNFα and IL-6. TNFα was significantly elevated for both cell types when iron-fed and IL-6 was significantly decreased. Iron-fed C8B4 cells also showed an elevated level of IL-1β, but decreased levels of IL-10. In contrast, iron-fed primary microglia showed decreased levels of IL-1β and increased levels of IL-10.

### 3.6. Conditioned Medium from Iron-Fed Microglia Caused Increased α-Synuclein Expression

**Figure 6:**
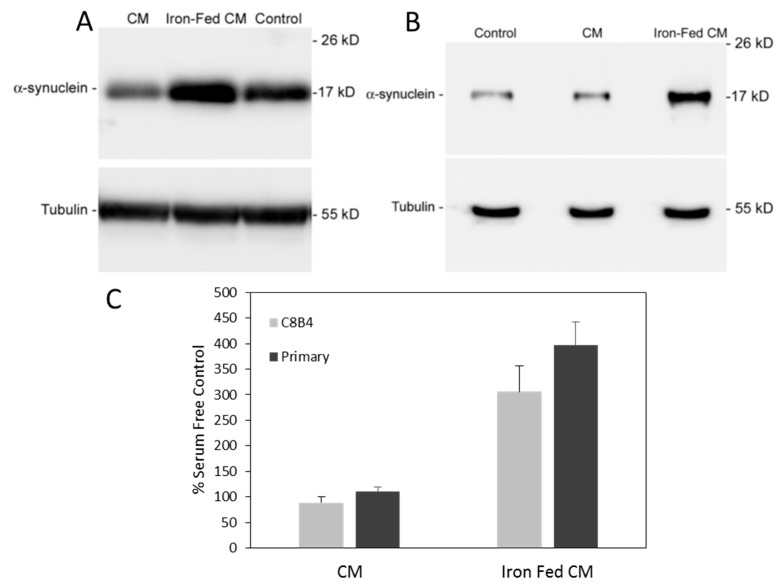
Increased levels of α-synuclein protein. SH-SY5Y (human neuroblastoma) cells were treated for 24 h with serum free medium (control) conditioned medium from control (CM) and iron-fed microglia (Iron-fed CM). The level of α-syn expressed by SH-SY5Y cells was assessed by western blot using a specific antibody. Protein loading levels were assessed using western blot for tubulin. The effect of conditioned medium from either (**A**) C8B4 or (**B**) Primary microglia was assessed and densitometric analysis carried out (**C**). Compared to SH-SY5Y cells grown in serum free medium (control) conditioned medium from control microglia (CM) had no significant effect (*p* > 0.05). In comparison, conditioned medium from iron-fed microglia (Iron-fed CM) caused a significant increase (*p* < 0.05) in the levels of α-syn protein detected. Conditioned medium from both C8B4 and primary microglia has a similar effect. Shown are the mean and S.E.M. of four separate experiments.

Changes in α-syn expression can easily be assessed using western blotting of protein extracts from cell lines, such as SH-SY5Y. Conditioned medium was generated from both control and iron-fed C8B4 microglia plated at equal density (40%). Iron-fed C8B4 cells had been grown in 500 µM ferric ammonium citrate for at least two weeks before use. The iron-fed cells were washed with fresh medium to remove trace iron before production of conditioned medium. Serum free medium (DMEM and B27 without antioxidants) was applied to the C8B4 cells and the conditioned media were collected after 48 h. The media were cleared of debris by centrifugation and filtered before application to SH-SY5Y cells. The conditioned media were applied to the SH-SY5Y cells for 24 h after which time protein extracts were prepared from the cells. The level of expression of α-syn was determined by western blotting and detection with a specific antibody to α-syn. Conditioned medium from control microglia had no effect on α-syn expression while conditioned medium from iron-fed microglia showed an approximate three-fold increase in the level of α-syn detected (Figure 6A). In comparison, 50 µM ferric ammonium citrate had no effect on α-syn expression (Supplementary Figure S1). Additionally, conditioned medium from the microglia had no effect on SH-SY5Y cell viability (Supplementary Figure S2).

As the effect on α-syn expression may have been related to the use of microglial cell lines we sought to confirm the effect by using primary mouse microglia isolated from new-born mice. Mixed glial cultures were prepared and treated with 500 µM ferric ammonium citrate for at least two weeks. Microglia were then isolated and purified from these cultures (along with controls that were not iron-fed). Conditioned medium was then generated from these cultures and applied to SH-SY5Y for 24 h. Conditioned medium from iron-fed microglia but not controls caused an increase in expression of α-syn, as assessed by western blotting (Figure 6B).

### 3.7. SNCA Promoter Activity Is Increased by Conditioned Medium from Iron-Fed Microglia

**Figure 7:**
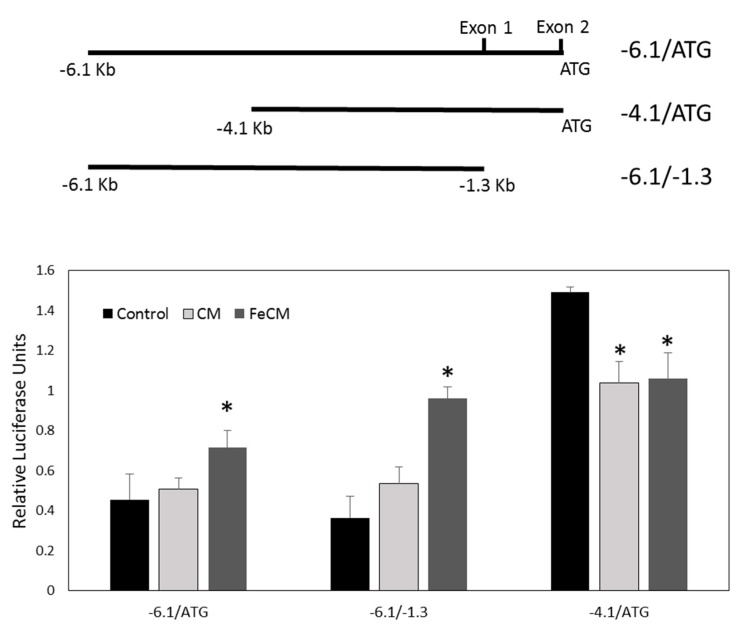
SNCA transcriptional activity. We wished to determine if the change in α-syn protein levels in cells treated with iron-fed microglial conditioned medium was due to a change in activity of the promoter of cytosolic protein alpha-synuclein (α-syn), SNCA. We used three reporter constructs containing fragments of the SNCA promoter that drive luciferase expression when transcriptionally active. The fragments are illustrated by the schematic which shows the overlap with the main features of the SNCA promoter. −6.1/ATG covers the majority of the promoter as well as the 5′ non-coding domain prior to the start codon. −6.1/−1.3 covers the same part of the promoter as −6.1/ATG but excludes the non-coding exons. The fragment −4.1/ATG excludes 2 kb of the sequence at the 5′ end of the promoter. SH-SY5Y cells were transiently transfected with the three constructs and the cells were treated for 24 h with either serum free medium (control), conditioned medium from C8B4 microglia (CM) or conditioned medium from iron-fed C8B4 microglia (FeCM). Readout of luciferase activity showed that conditioned medium from C8B4 microglia had no significant effect on the −6.1/ATG or −6.1/−1.3 promoter fragment, while iron-fed conditioned medium caused a significant increase (*p* < 0.05) in activity. In contrast the conditioned medium from both kinds of microglia decreased the activity seen with the −4.1/ATG fragment. The difference in activity suggests that the change in expression of α-syn may come from binding of a transcription factor between −6.1 and −4.1 on the SNCA promoter in response to a factor in iron-fed microglia conditioned medium. Shown are the mean and S.E.M. of four separate experiments.

Change in α-syn expression may come from either increased transcription or decreased breakdown of the protein. The first step in assessing this process is to assay transcription of the protein from its gene. In order to do this, we used three luciferase reporter constructs based on the α-syn SNCA promoter [31]. The largest of these promoter fragments was 6.1 kb in size. Its 3′ end coincided with the start codon of the α-syn ORF and was termed −6.1/ATG (Figure 7). Two further constructs included smaller regions of this fragment. −6.1/−1.3 was missing a large 3′ section prior to the start codon and −4.1/ATG was missing 2 kb from the 5′ end. The fragments were cloned into a firefly luciferase expression vector and transiently transfected in SH-SY5Y cells in parallel with a renilla luciferase expressing plasmid driven by the thymidine kinase promoter (pTK) to control for differences in cell number and transfection efficiency. 24 h after transfection, the cells were treated with conditioned medium either from control or iron-fed C8B4 microglia plated at equivalent density. After 24 h a dual luciferase assay was performed on extracts from the SH-SY5Y cells. A significant increase in luciferase activity was seen with the reporters −6.1/ATG and −6.1/−1.3 when SH-SY5Y cells were treated with conditioned medium from iron-fed microglia (Figure 7). No change was observed when conditioned medium from control microglia was used. The smaller fragment −4.1/ATG showed a significant decrease in reporter activity when conditioned medium from both control and iron-fed microglia was applied. The response of this smaller fragment did not explain the differences in protein expression observed when conditioned medium from iron-fed microglia was applied to SH-SY5Y cells, whereas the response of the −6.1/−1.3 reporter fragment matched the increase. These results suggest that the increase in α-syn expression may be induced at the transcriptional level and it involve a transcription factor that binds between −6.1 and −4.1 in the SNCA promoter.

### 3.8. Neutralisation of Cytokines Released from Iron-Fed Microglia Blocks Increased α-Synuclein Expression

**Figure 8:**
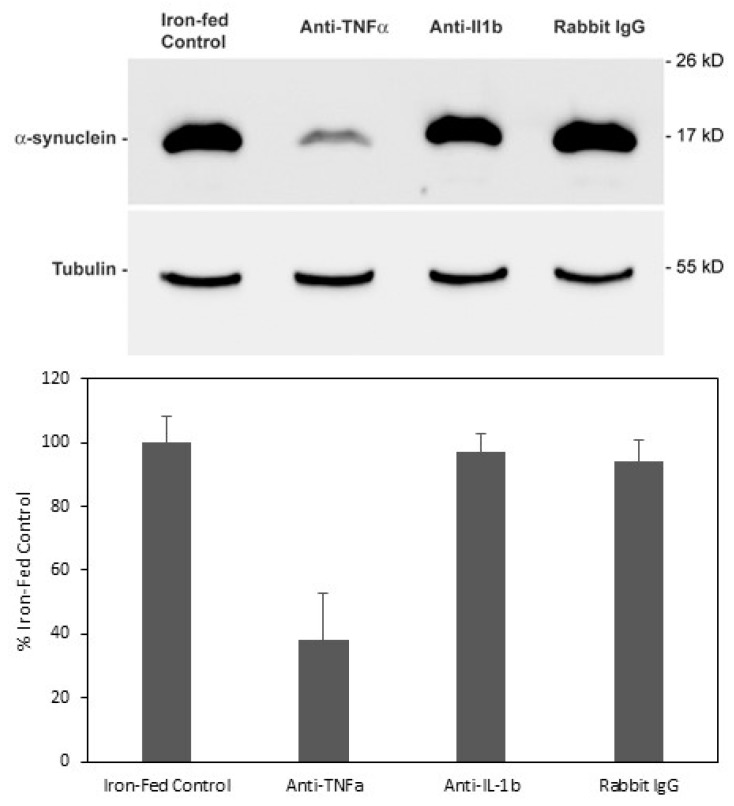
Cytokine Neutralization SH-SY5Y. Cells were treated with conditioned medium from iron-fed C8B4 microglia pre-treated with antibodies in an attempt to neutralize cytokines present that may be responsible for inducing increased expression of α-syn. We tested the effect of antibodies to TNFα, IL-1β and as a control, rabbit IgG. Medium was treated with 150 ng/mL of the specific antibody for one hour before applying to the SH-SY5Y cells. After 24 h western blot was used to assess the level of α-syn protein and tubulin to compare loading. On the antibodies tested only anti- TNFα caused a significant (*p* < 0.05) reduction in the level of α-syn detected. Shown are the mean and S.E.M. of four separate experiments.

We measured changes in the level of cytokines that is released by iron-fed microglia, as indicated above. The most consistent change was in TNFα. There was also an increase in IL-1β for C8B4 microglia. Decreased cytokine levels were considered of no interest, as conditioned medium from control microglia had no effect on α-syn levels. Therefore, we attempted to neutralize the increase in α-syn that occurred in SH-SY5Y cells in response to treatment with conditioned medium from iron-fed microglia. Conditioned medium from iron-fed microglia was applied to SH-SY5Y cells as before. Some samples of conditioned medium were pre-treated with either an antibody to TNFα, IL-1β, or an anti-rabbit IgG (150 ng/mL). After 24 h, the level of α-syn protein in the cells was determined by western blot. Only neutralization with the TNFα antibody resulted in the reduction in measured α-syn levels (Figure 8). This implies that the increased level of α-syn induced by iron-fed conditioned medium is possibly caused by TNFα.

**Figure 9:**
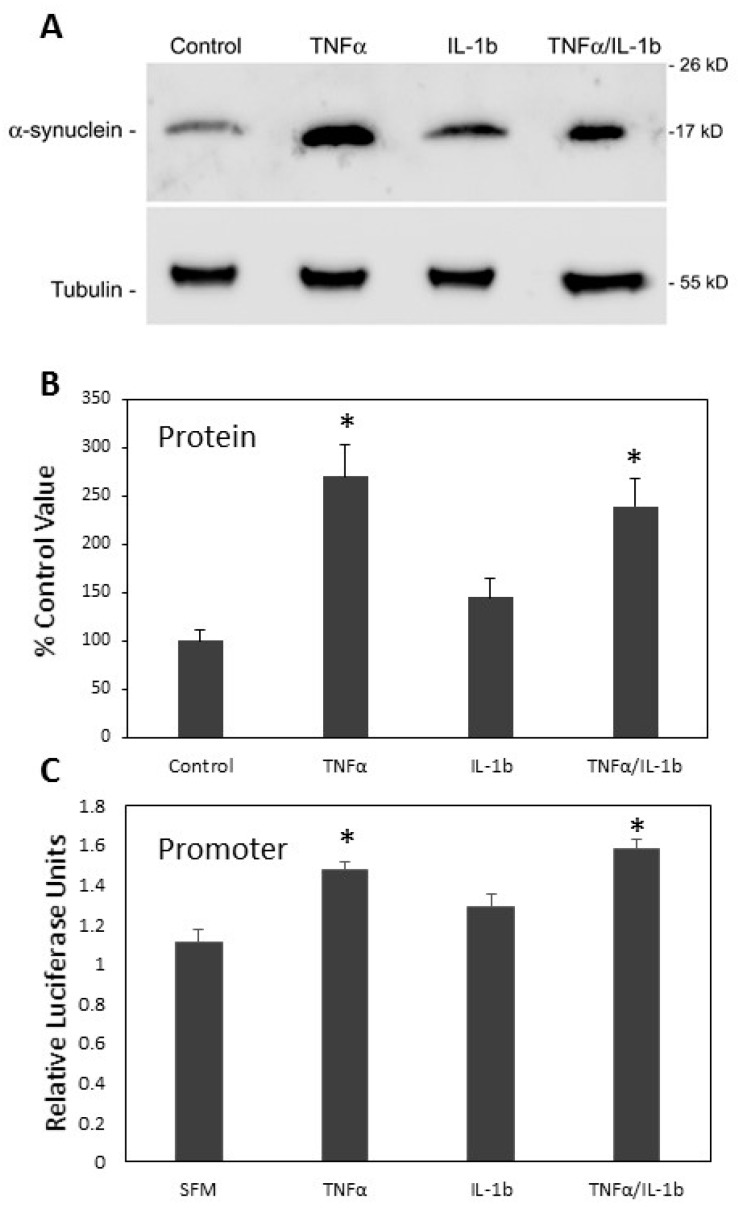
Cytokines and α-synuclein expression. (**A**) We tested cytokines to determine if they could induce increased expression of α-syn in SH-SY5Y cells. SH-SY5Y cells were grown in serum free medium and 50 ng/mL of either mouse TNFα, IL-1β, or both was applied to the cells twice in a 24 h period. Western blot was then carried out to assess α-syn expression levels. Tubulin was also assessed as a loading control. (**B**) Analysis showed that only TNFα or TNFα/IL-1β caused an increase in α-syn expression. As IL-1β had no significant effect this result was only due to the presence of TNFα. (**C**) We also tested the effects of the cytokines on the activity of the SNCA promoter reporter −6.1/−1.3 which showed the strongest response to iron-fed conditioned medium. The SH-SY5Y cells transiently transfected with the reporter were treated similarly with the cytokines. Luciferase activity was significantly (*p* < 0.05) increased only for cells treated with TNFα alone or in combination with IL-1β. Shown are the mean and S.E.M. of four separate experiments.

We tested whether TNFα or IL-1β could increase the expression of α-syn in SH-SY5Y cells. SH-SY5Y cells were treated with 50 nM TNFα, 50 nM IL-1β, or both in serum free medium supplemented with B27. After 24 h the levels of α-syn were assessed by western blot. Only TNFα caused a significant increase in α-syn levels (Figure 9A,B). The combination of TNFα and IL-1β was not significantly different to TNFα alone. We also tested the effects of the same cytokines on the activity of the SNCA promoter fragment −6.1/−1.3. The luciferase reporter fragment was transfected into SH-SY5Y cells and treated with 50 nM TNFα, 50 nM IL-1β, or both in serum free medium supplemented with B27. The measured luciferase activity showed a significant increase in the promoter activity in SH-SY5Y cells when treated with TNFα but not IL-1β (Figure 9C). These results combined suggest that the molecule released from iron-fed microglia that increased α-syn expression is TNFα. 

### 3.9. Increased α-Synuclein Expression Induced by Iron-Fed Microglia Was Mediated by the NF-κB Pathway 

**Figure 10:**
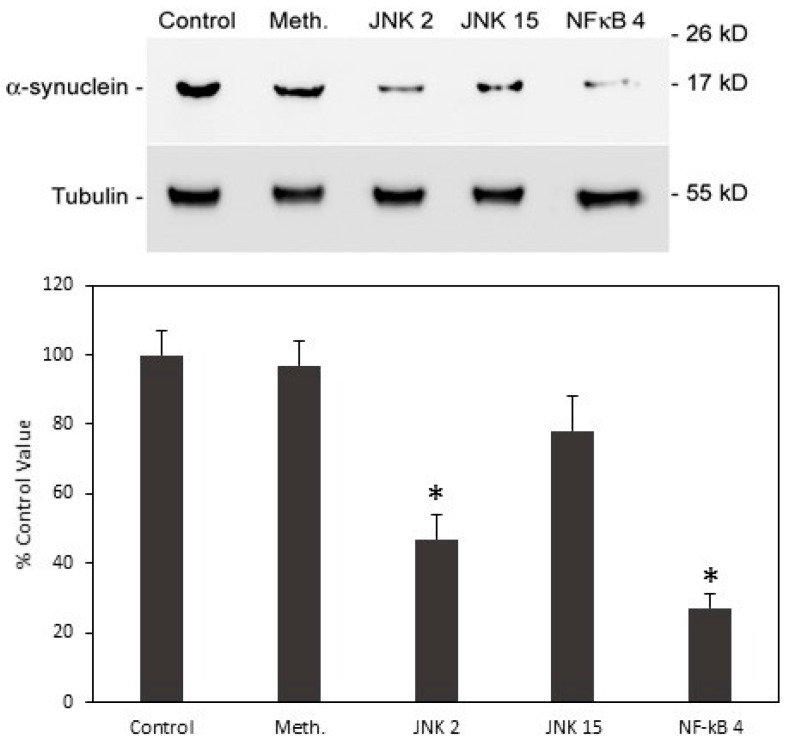
Inhibitors of signaling pathways. In order to assess the role of both the JNK pathway and NF-κB pathway in the increased expression of α-syn in response to conditioned medium from iron-fed C8B4 microglia, we applied a series of inhibitors of these pathways to SH-SY5Y cells during treatment with the conditioned medium. The compounds used were two inhibitors of the JNK pathways (JNK 2 and JNK 15) and one inhibitor of the NF-κB pathway (NF-κB 4). As the inhibitors were soluble in methanol we also included a methanol control at the highest volume used. After a 24 h treatment, the levels of α-syn and tubulin were assessed by western blot. The strongest inhibition was seen with the NF-κB 4 inhibitor. The inhibitory effect of JNK 2 was also significant (*p* < 0.05) but the effect of JNK 15 was not. Shown are the mean and S.E.M. of four separate experiments.

The two predominant pathways that are involved in signalling to the nucleus via TNFα occur through either JNK(c-Jun N-terminal kinase)/AP-1 or NF-κB (nuclear factor kappa-B). Potentially, increased α-syn expression that is caused by the conditioned medium from iron-fed microglia could be mediated through either pathway (or both). We used inhibitors of both JNK and NF-κB to determine whether blocking these pathways could prevent the increase in α-syn that is caused by the conditioned medium. 200 nM JNK inhibitor II (CAS129-56-6) [34], 25 μM JNK inhibitor XV (IQ-1S, CAS1421610-21-0) [35], and 300 nM NF-κB inhibitor IV (CAS 139141-12-1) [36] were applied to SH-SY5Y cells treated with C8B4 iron-fed conditioned medium for 24 h. The concentrations used were based on concentrations used effectively in the cited papers. After western blotting and detection of α-syn, the strongest inhibitory effect on α-syn expression was seen with the NF-κB inhibitor IV (Figure 10). While significant inhibition was also seen with the JNK inhibitor XV, there was no significant inhibition with JNK inhibitor II. These results suggest that the increased expression of α-syn induced by the iron-fed microglia conditioned medium is mediated through NF-κB but with the possibility that some of the effect is also mediated by the JNK pathway.

### 3.10. Conditioned Medium from Iron-Fed Microglia Caused a Decrease in α-Synuclein Activity

**Figure 11:**
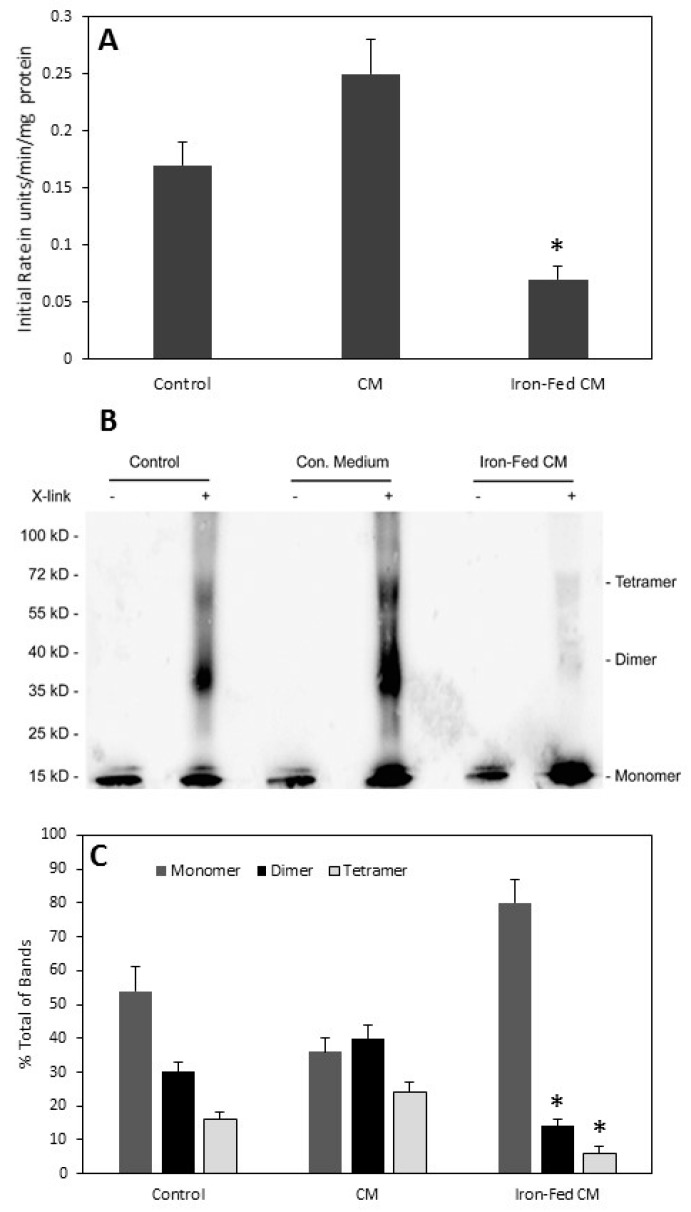
Ferrireductase activity and tetramers. (**A**) α-syn possesses ferrireductase activity and SH-SY5Y cells overexpressing α-syn show increased levels of iron reduction. We measured the ferrireductase activity in SH-SY5Y cells stably overexpressing α-syn. The cells were exposed to conditioned medium from either control C8B4 microglia or iron-fed microglia for 24 h. Protein extracts were made and the initial rate determined for a concentration of 500 µM ferric ammonium citrate using a standard ferrireductase assay based on colour change associated with the binding of Fe(II) to ferrozine. The results show that conditioned medium from iron-fed microglia significantly (*p* < 0.05) decreased measured ferrireductase activity while that from control microglia did not. Shown are the mean and s.e. of four separate experiments. (**B**,**C**) α-syn ferrireductase activity has been shown to be due to the presence of tetramers of α-syn in the cell membrane. We therefore measured the presence of α-syn tetramers in membrane extracts of α-syn overexpressing SH-SY5Y cells. Protein extracts were prepared from membrane fractions and cross-linked with DSS. Western blot was then used to assess the presence of monomers, dimers and tetramers of α-syn in the extracts. While treatment with C8B4 conditioned medium had no effect on the ratio of tetramers to dimers and monomers, treatment with conditioned medium from iron-fed C8B4 microglia caused a significant (*p* < 0.05) reduction in the levels of both tetramers and dimers. Shown are the mean and S.E.M. of four separate experiments.

We have shown previously that α-syn is able to reduce iron through ferrireductase activity [37]. This activity is present in vivo [38] and the active form of the protein is a tetramer [29]. Tetrameric α-syn has also been suggested to be the native form of α-syn [39] and its loss from the cell might lead to the formation of disease specific oligomers [40]. We analysed ferrireductase activity in cells overexpressing α-syn when exposed to the conditioned medium from iron-fed microglia. Conditioned medium from C8B4 control microglia had no significant effect on the measured ferrireductase activity (Figure 11), whereas conditioned medium from iron-fed microglia caused a significant reduction in the activity measured. As ferrireductase activity is associated with the tetrameric form of the protein we also assessed whether there was a change in the level of tetramers present in α-syn overexpressing cells that are exposed to conditioned medium from iron-fed microglia. α-syn tetramers can be identified in SH-SY5Y cells by cross-linking protein extracts from the cells. Tetramers can then be observed by western blot. SH-SY5Ys that are treated with conditioned medium from iron-fed microglia showed significantly reduced levels of tetramers when compared to cells treated with conditioned medium from control microglia (Figure 11). This data suggests that conditioned medium from iron-fed microglia reduced the formation of α-syn tetramers, and consequentially reduced the ferrireductase activity of α-syn measured in SH-SY5Y cells.

### 3.11. Aggregation of α-Synuclein

**Figure 12:**
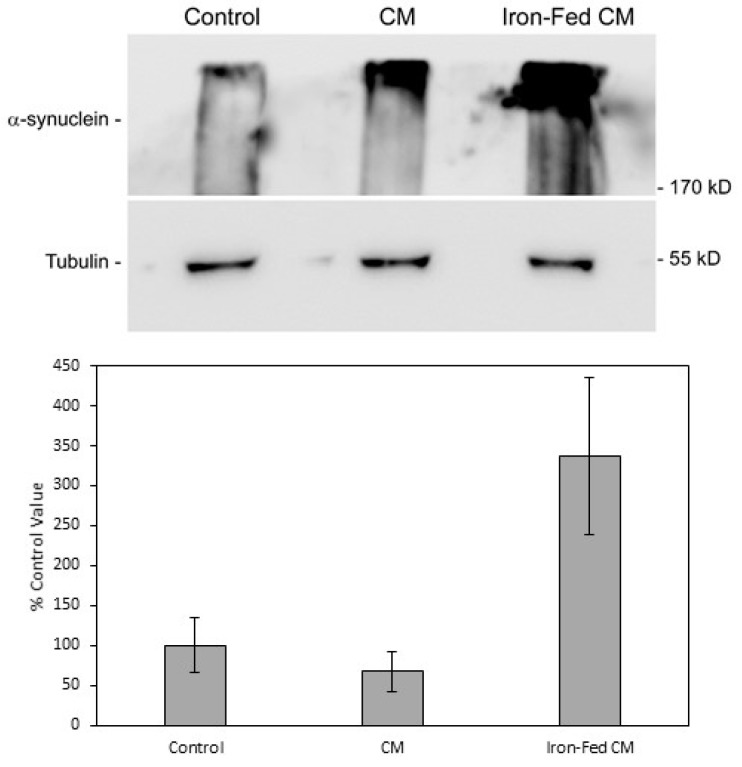
Aggregation of α-syn. Western blot was used to detect high molecular weight aggregates of α-syn in extracts from SH-SY5Y cells treated with conditioned medium from primary microglia. SH-SY5Y cells were treated for 24 h with medium from control microglia (CM) or iron-fed microglia (Iron-Fed CM). Some cells were treated with just serum free medium (control). Extracts were prepared and electrophoresed on a 6% PAGE gel. The protein was then transferred by blot to a PVDF membrane (3 h, 100 mA) and α-syn detected with a specific antibody. High molecular weight bands for α-syn were indicative of aggregates. We also verified protein loading by re-probing the same blots for tubulin. Bands for α-syn were then analysed densitometrically. Values for control were normalised to 100% and values for the treated samples compared. Only treatment with iron-fed conditioned medium caused a significant (*p* < 0.05) increase in α-syn detected in the aggregate band. Shown are the mean and S.E.M. for four separate experiments.

The aggregation of α-syn into oligomeric species is considered to be a major hallmark of diseases that are associated with α-syn. Aggregates of α-syn can frequently be detected in cells overexpressing the protein. We had previously developed a western blot assay to detect oligomeric aggregates of α-syn [26]. SH-SY5Y cells overexpressing α-syn were treated with conditioned medium from primary control microglia or iron-fed microglia for 24 h. Extracts were prepared from the SH-SY5Y cells and electrophoresed on a PAGE gel. After western blot and detection with a specific antibody, high molecular weight (>300 kD) bands of α-syn oligomers were detected. Treatment with control microglia conditioned medium had no significant effect on the levels of aggregates detected (despite a high level of variability). However, treatment with conditioned medium from iron-fed microglia results in a large and significant increase in the level of aggregates detected (Figure 12). This implies that conditioned medium from iron-fed microglia is able to induce the aggregation of α-syn independently of its effect on protein expression. A similar result was observed with conditioned medium from C8B4 cells (data not shown).

## 4. Discussion

The aim of the work presented here was to establish a model of microglia that could be used to replicate aspects of the aging brain. The study of both normal aging and neurodegenerative diseases is compromised by the lack of effective models of the aging brain. Neurodegenerative diseases are predominantly associated with aging, as it is considered the single most important risk factor for their development. However, studies rarely ever incorporate conditions related to the aging phenotype. This is particularly hampered by poor definition of exactly what changes are important to consider. Numerous reports described changes in oxidative stress [41], by-products of oxidative damage [42], or trace metals that can themselves induce oxidative stress and subsequent by-products [43]. Here, we have utilized knowledge of the phenotype of dystrophic microglia to modify a microglial cell line and combine it with the study of disease-associated changes in α-syn.

Developing a model of senescent/dystrophic microglia in vitro has numerous issues. Chief among these is the lack of clarity in defining dystrophic microglia [13]. There is currently no single molecular marker that would define a dystrophic or senescent microglial cell. Proteomics/transcriptomics based studies comparing microglia from old and young brains have been carried out for both mouse and human but have yielded conflicting results [44,45,46,47]. However, there is a general cellular senescence signature that all cells show and this is no different for microglia, which also show characteristics aligning with a senescence-associated secretory phenotype [48]. 

The second hurdle for generating an in vitro model is the difficulty in using primary microglia. Isolated primary microglia rapidly change phenotype [49,50,51], microglia from adult mice are difficult to maintain [52] (there are still very few studies using them), and pushing microglia into a senescent/dystrophic phenotype reduces both their yield and viability. For these reasons, we predominantly used a microglial cell line as this allowed for us to generate large numbers of microglia with our iron-fed phenotype.

Dystrophic microglia in the aging brain do have a number of clear differences to normal resting microglia. These include morphological changes, such as loss of processes, increased iron storage, and increased expression of ferritin [9,10,13,53,54,55]. Of considerable interest to us was the evidence for increased iron storage. While in a normal aging situation the accumulation of iron is likely a consequence of the aging process, iron is highly associated with the risk of oxidative damage, a hallmark of aging. Therefore, a possibility exists that increasing iron storage experimentally would induce the changes observed in dystrophic microglia in vivo. There is already considerable evidence that iron-overload can induce senescent changes in cells including microglia [56,57,58]. In this light, our finding that cultured microglia maintained in a high iron environment adopt characteristics of dystrophic microglia is not surprising. 

As our iron-fed microglia are a model of dystrophic microglia they also demonstrate characteristics of the senescence-associated secretory phenotype (SASP) referred to previously. This includes the reduced proliferation and increased release of pro-inflammatory cytokines such as TNFα. A recent study has suggested that the potassium channel Kv1.3 is increased in expression in aged microglia [33]. This was a finding we also confirmed in our iron-fed microglia. Further study from the same group showed that in aged mice, Kv1.3 was associated with release of pro-inflammatory cytokines including TNFα [59]. Therefore, increased expression of Kv1.3 and its subsequent down-stream effects are likely a part of SASP.

As mentioned above, our aim was to develop a model of aged microglia that could be applied to the study of neurodegenerative diseases. Microglia and also dystrophic microglia have been implicated in many neurodegenerative diseases [5,55,60]. We chose to concentrate on synucleinopathies on the basis of our previous experience studying the role of α-syn in disease models. There is strong evidence that microglia play a role in synucleinopathies, such as PD [61], multiple system atrophy [62], and Dementia with Lewy Bodies [24,63]. There is currently also considerable interest in the impact of age-related changes to microglia in PD [8,64] and it has even been suggested to be causative of neurodegeneration in the *substantia nigra* [23,65].

Synucleinopathies are associated with the aggregation of α-syn in cells and this is believed to stem from two causative processes. The first and most well recognized is an increased expression of α-syn, resulting in molecular crowding [66,67]. The second and more controversial is the more recent suggestion that the native and functional form of α-syn is a tetramer and loss of tetramer formation increases the likelihood of aggregation [40]. Using conditioned medium from our model dystrophic microglia, we were able to induce increased α-syn expression, reduced tetramer formation and increased aggregation in SH-SY5Y cells. Thus, by the incorporation of an aspect of brain aging we were able to induce several aspects of the disease state in neuronal cells. For this reason, we believe that we have developed a simple and valuable tool for the exploration of the molecular mechanisms behind synuclein related diseases and possibly other neurodegenerative diseases.

The ability of conditioned medium from iron-fed microglia to induce changes in α-syn in SH-SY5Y cells implies that a soluble factor released by the microglia is responsible. We showed that the levels of iron released from the iron-fed microglia are small and application of iron to SH-SY5Y does not cause the same response. There is also no evidence in the literature that iron levels alter α-syn transcription. In contrast, we were able to show that iron-fed microglia release increased levels of TNFα, neutralization of TNFα blocks the increased α-syn expression and that exogenous TNFα also induced increased α-syn expression and transcription. This implies that TNFα mediates the effects we observed. This result is further supported by findings showing that an inhibitor of NF-κB blocks the increased expression as well. TNFα effects on protein expression via transcriptional activation are frequently mediated through an NF-κB controlled pathway [68]. There have been few papers linking TNFα and α-syn, but one paper has shown that TNFα increases α-syn in control human iPS cells [69]. Another paper did suggest that TNFα released by microglia could impair autophagic flux and this increased α-syn levels through decreased breakdown of the protein [70]. It is also possible that decreased autophagy could contribute to the increased levels of α-syn that we observed. Similarly, there been very few reports that α-syn expression can be increased by NF-κB, even though it has been reported there are NF-κB binding sites on SNCA [69]. Our data showed that there is a fragment of the SNCA promoter that appears to be necessary for TNFα-driven induction of expression (between −6.1 and −4.1 kb) and analysis with online transcription factor binding site software (Match, Biobase, Germany) identified at least three potential binding sites in this region for NF-κB. Analysis of patients with PD has shown increased TNFα levels and increased nuclear localisation of NF-κB in neurons and microglia in the substantia nigra [71]. In general, there is a significant amount of data implying that NF-κB activation might be relevant to PD [72,73,74]. This suggests our model might have relevance to the changes observed in PD.

TNFα is not the only cytokine that is altered in Parkinson’s disease [75]. Many studies look at cytokines that are released by microglia in response to α-syn rather than changes in microglial released cytokines that could alter neuronal activity in PD [76]. In this regard, aggregates of α-syn induced neurotoxic effects that are mediated by microglia through the activation of Toll-like receptor 2 [77]. We also observed other changes in cytokines other than TNFα. However, these appeared to have no impact on α-syn expression such as IL-1β or were higher in control microglia than the iron-fed ones. In this case, as we saw no change in α-syn expression when treated with control conditioned medium versus untreated, the levels of these cytokines, regardless of how they changed, had no impact on α-syn. While the microglia that we used were murine in origin and the SH-SY5Y cells were human, most murine cytokines are able to bind to human cytokine receptors, the most notable exception being IL-10 where the murine form cannot bind to the human IL-10 receptor [78].

We noted other changes in α-syn that we have not linked to TNFα. These include tetramer formation and ferrireductase activity. However, the regulation of both of these aspects of α-syn activity is currently unknown. We have previously shown that tetramer formation is connected to increased ferrireductase activity, while increased aggregation is linked to a loss of this activity [29,38]. The process of aggregation of α-syn is likely to require the protein to initially pass through small oligomeric states. However, the stable tetramer that is expressed in cells is supposed to be highly structured with a helical configuration and its formation is suggested to prevent higher order oligomerization and aggregation [39,79]. We also observed that conditioned medium from iron-fed microglia induced increased levels of aggregation. Aggregation of α-syn into potentially toxic oligomeric species is considered to be one of the hallmarks of synucleinopathies [80,81]. As this is accompanied by a reduction in tetramer formation, this change possibly represents the mechanism by which aggregation could occur in diseases, like PD. This model system therefore may be of benefit in analysing the mechanics of conversion of α-syn from its normal cellular isoform to the oligomeric aggregates generated in disease.

## 5. Conclusions

In summary, we have created a model to incorporate an aspect of brain aging into the study of α-syn. This model is able to recapitulate a number of changes that are observed in diseases, like Parkinson’s disease. These changes include increased expression, aggregation, reduced tetramer formation and ferrireductase activity of α-syn. The model incorporates a potential in vitro dystrophic microglia component. By overloading microglia with iron, we have shown that they behave similarly to dystrophic microglia, altering their morphology, iron storage, protein expression and cytokine release. The utility of these model dystrophic/senescent microglia will allow for further study of both the ontology of dystrophic microglia and the potential role of such microglia in neurodegenerative disease. This may allow for more robust in vitro models for the study of these complex diseases.

## Figures and Tables

**Table 1:** Cytokine released into conditioned medium.

	C8B4 Microglia		Primary Mouse Microglia	
Cytokine (ng/mg)	Control	Iron-Fed	Control	Iron-Fed
TNFα	142.82 ± 12.40	192.46 ± 5.52 *	37.42 ± 3.74	84.19 ± 3.45 *
IFNγ	n.d.	n.d.	n.d.	n.d.
KC/GRO	n.d.	n.d.	698 ± 16	2113 ± 398 *
IL-1b	2.93 ± 0.27	3.83 ± 0.22 *	0.99 ± 0.08	0.34 ± 0.03 *
IL-2	n.d.	n.d.	n.d.	n.d.
IL-4	n.d.	n.d.	n.d.	n.d.
IL-5	n.d.	n.d.	n.d.	n.d.
IL-6	490.96 ± 49.77	430.77 ± 18.05	356.09 ±4 7.84	117.56 ± 24.22 *
IL-8	n.d.	n.d.	n.d.	n.d.
IL-10	6.20 ± 1.04	0.84 ± 0.26 *	5.00 ± 0.37	10.34 ± 0.98 *
IL-12p70	n.d.	n.d.	n.d.	n.d.
IL-13	n.d.	n.d.	n.d.	n.d.

Serum free conditioned medium was generated from cultured microglia over 24 h and the levels of cytokines assessed with the Meso Scale Discovery (MSD) ELISA system. Cytokine concentrations were determined by comparison to a standard curve for each cytokine. Values were the concentration in the medium (ng/mL) divided by the concentration of the protein in the cells that were used to generate the conditioned medium (mg/mL). Shown are the mean (ng/mg) and S.E.M. for five (C8B4) and three (primary) different experiments. * Indicates a significant difference between control and iron-fed (*p* < 0.05). n.d. indicates not detectible due to the levels detected being below the level of lowest standard on the standard curve for that cytokine.

## Supplementary Materials

The following are available online at http://www.mdpi.com/2218-273X/8/3/67/s1. Supplementary Figure S1 Iron and α-synuclein expression, Supplementary Figure S2 Toxicity of Conditioned Medium.
