# Supplementary material for: Model Senescent Microglia Induce Disease Related Changes in α-Synuclein Expression and Activity
Source: Biomolecules. 2018 Aug 1;8(3):67. doi: 10.3390/biom8030067 (PMC6164966; doi:10.3390/biom8030067)
Supplement: Supplementary file 1 [file biomolecules-08-00067-s001.pdf]

biomolecules

Supplementary Data

Model Senescent Microglia Induce Disease Related Changes in Alpha-synuclein Expression and Activity.

Dafina M. Angelova and David R. Brown

Department of Biology and Biochemistry, University of Bath, BA2 7AY, Bath, UK.

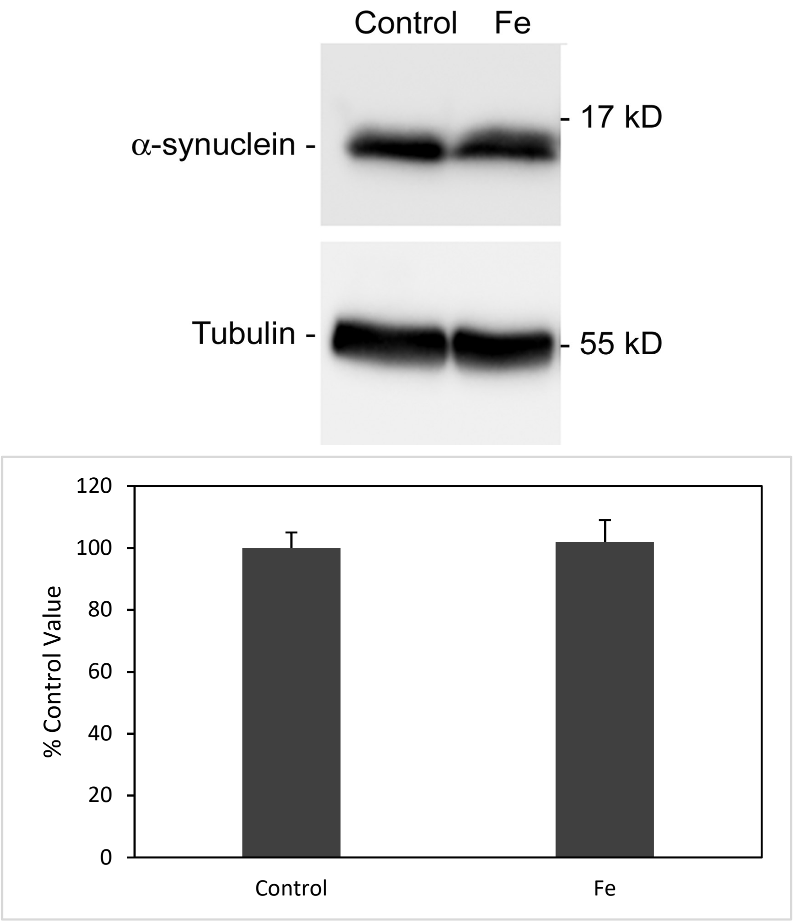

Supplementary Figure 1 Iron and α-synuclein expression.

SH-SY5Y cells were grown in serum free medium for 24 h. Control cells were grown only in the serum free medium while iron treated cells (Fe) were grown in 50 μM ferric ammonium citrate. After 24 h the cells were harvested, and protein extracted. Equal amounts of protein were eletrophoresed on a 14% PAGEgel. After semi-dry transfer to a membrane α-syn was detected with a specific antibody (MJFR1) and bands detected with chemiluminescence. The detected bands were quantitated densitometrically. The process was repeated after stripping the blot and tubulin was detected with a monoclonal antibody. Treatment with iron had no effect on the levels α-syn detected. Shown are the mean and S.E.M for four experiments.

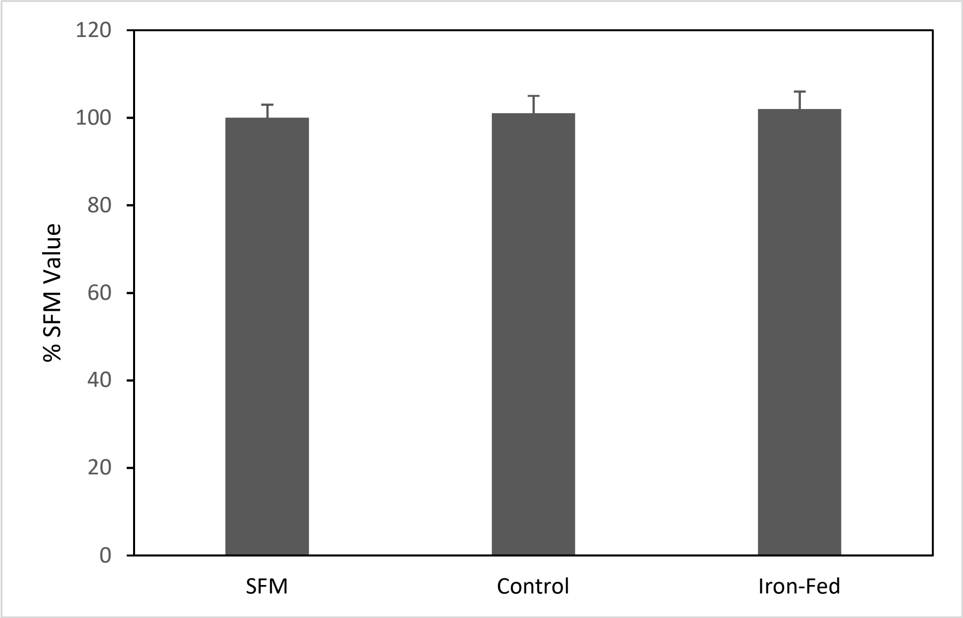

Supplementary Figure 2: Toxicity of conditioned medium.

The toxicity of conditioned medium from C8B4 microglia was tested on SH-SY5Y cells. The cells were treated for 24 h with either serum free medium (SFM), conditioned medium from control microglia (Control) or iron-fed microglia (Iron-Fed). The survival after 24 h was determined using an MTT viability assay. Neither the medium from control microglia nor iron-fed microglia had any significant ($p > 0.05$) effect on SH-SY5Y cell viability when compared to SFM. Shown are the mean and S.E.M for four separate experiments with three replicates each.

© 2018 by the authors. Submitted for possible open access publication under the terms and conditions of the Creative Commons Attribution (CC BY) license (http://creativecommons.org/licenses/by/4.0/).
